# Bibliometric analysis of inflammatory bowel disease and emotional factors: trends, impact, and emerging research areas

**DOI:** 10.1097/MS9.0000000000003982

**Published:** 2025-10-10

**Authors:** Mehrnaz Asgharnezhad, Pegah Rashidian, Negin Letafatkar, Abdulhadi Alotaibi, Mohanad Khalid Alfarra, Omar Hasan Hasan, Saman Maroufizadeh, Narges Soltani, Reyhane Pasandipour, Farahnaz Joukar, Fariborz Mansour-Ghanaei

**Affiliations:** a Gastrointestinal and Liver Diseases Research Center, Guilan University of Medical Sciences, Rasht, Iran; b Department of Medicine and Surgery, Vision Colleges, Riyadh, Saudi Arabia; c School of Medicine, Hormozgan University of Medical Sciences, Bandar Abbas, Iran

**Keywords:** anxiety, bibliometric analysis, depression, emotional factors, inflammatory bowel disease, psychological care, stress

## Abstract

**Background:**

Inflammatory bowel disease (IBD), encompassing Crohn’s disease and ulcerative colitis, is a chronic gastrointestinal condition influenced by genetic, environmental, and immunological factors. Emerging evidence underscores the significant role of emotional factors in the onset, progression, and management of IBD. This bidirectional relationship necessitates a multidisciplinary approach that integrates psychological care into IBD management strategies. This study employs bibliometric analysis to provide a comprehensive overview of the research landscape on IBD and emotional factors.

**Methods:**

We used the Web of Science Core Collection to search for pertinent publications. To conduct the analyses, we utilized tools like VOS Viewer, CiteSpace, and Biblioshiny.

**Results:**

Research in this field has shown exponential growth, with annual publications increasing from fewer than five in the 1980s to 193 in 2023. The United States leads in research output (521 publications) and collaboration centrality (0.72), followed by England and Canada. The University of Manitoba is the top contributing institution, and Charles N. Bernstein emerged as the most prolific author. Journals like *Journal of Crohn’s & Colitis* and *Inflammatory Bowel Diseases* were pivotal in disseminating research. Cocitation analysis revealed Antonina Mikocka-Walus and Charles N. Bernstein as influential contributors to the field.

**Conclusion:**

The field of IBD and emotional factors is experiencing rapid growth, driven by increasing recognition of the psychological dimensions of IBD management. While significant progress has been made, gaps remain in understanding the underlying mechanisms and developing integrative therapeutic approaches. Future research should focus on longitudinal studies, interdisciplinary collaboration, and the incorporation of emotional health into personalized treatment strategies.

## Introduction

Inflammatory bowel disease (IBD), encompassing conditions such as Crohn’s disease and ulcerative colitis, is a chronic, relapsing, and debilitating disorder of the gastrointestinal tract affecting millions worldwide^[1]^. Characterized by persistent inflammation, abdominal pain, diarrhea, and systemic complications that impair patients’ quality of life^[2]^. IBD has a multifactorial etiology, involving genetic, environmental, and immunological factors^[3–5]^. However, growing evidence highlights the critical role of emotional and psychological factors in the onset, progression, and management of IBD^[3,6]^. Emotional factors are not only potential triggers for disease exacerbations but also impact treatment adherence and clinical outcomes^[6,7]^. Additionally, studies have shown that patients with IBD have a significantly higher risk of developing anxiety and depression compared to the general population^[8]^. Mood disorders, including anxiety, depression, and stress, have been shown to influence disease activity, treatment outcomes, and patients’ quality of life^[3,9]^. The bidirectional relationship between psychological distress and disease progression highlights the necessity of integrating psychological care into IBD management^[10]^. This intersection of gastroenterology and psychology has garnered increasing interest, as it offers a holistic approach to understanding and managing IBD.

**HIGHLIGHTS**

- Despite increasing evidence linking emotional factors to inflammatory bowel disease (IBD), no comprehensive bibliometric study has mapped the evolution and focus of this interdisciplinary field.
- The field has seen rapid growth since 2015, with anxiety, depression, and quality of life emerging as dominant research hotspots in IBD-related emotional studies.
- Future research should prioritize integrative, multidisciplinary approaches and explore emotional interventions to improve patient outcomes in IBD.

Despite the burgeoning interest, the research landscape surrounding IBD and emotional factors is complex and fragmented. Studies vary widely in their focus, methodologies, and geographical scope, making it challenging to discern overarching trends and breakthrough findings. Bibliometric analysis, a method that quantitatively evaluates the impact, scope, and trends of scientific publications, provides a powerful tool to synthesize this growing body of literature.

As bibliometric analysis has proven to be a valuable tool for identifying influential publications and research patterns in various subfields of gastroenterology^[11–13]^, this paper aims to offer a comprehensive overview of the research landscape on IBD and emotional factors through a bibliometric lens. By analyzing publication trends, influential studies, and collaborative networks, this study seeks to highlight key breakthroughs, identify research gaps, and propose future directions. Such insights are crucial for researchers, clinicians, and policymakers aiming to advance the understanding and management of IBD in the context of emotional health.

## Methods

### Data collection

**Figure 1.**
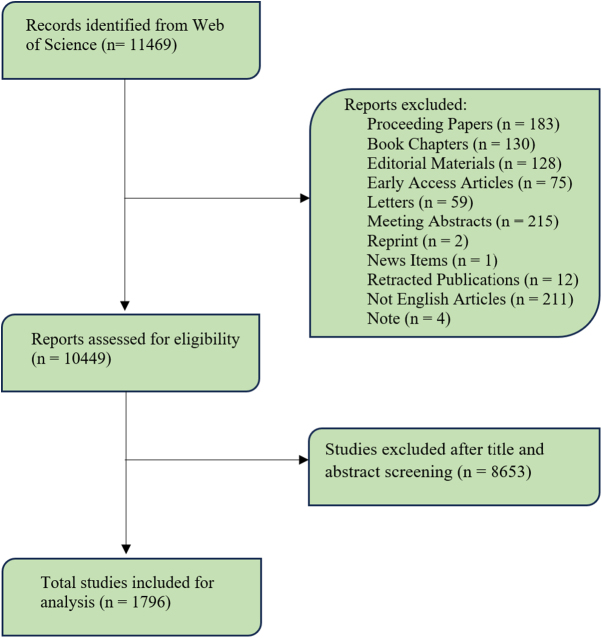
Study selection process.

To gather relevant articles for this study, we conducted a comprehensive search on the Web of Science Core Collection on 20 December 2024. This prestigious database, which houses over 12 000 reliable academic articles, is renowned for its breadth and quality of scientific content, making it an ideal resource for bibliometric analysis^[14–16]^. We devised a robust search strategy, incorporating a series of carefully selected keywords to maximize the precision and relevance of our results, as outlined in Supplemental Digital Content Table S1, available at: http://links.lww.com/MS9/A977. Initially, we retrieved 11 469. records from the database. To refine our selection and ensure alignment with the research objectives, we applied stringent inclusion and exclusion criteria. We excluded unrelated studies, such as book chapters, editorials, conference papers, letters, and prepublication articles, resulting in a final set of 1796 studies (Fig. 1). This selective approach helped ensure that the dataset accurately represented the scope of our investigation.

### Data analysis

For the analysis of the collected articles, we employed three advanced tools: VOSviewer, CiteSpace, and Biblioshiny (R Bibliometrix package). These software tools are widely used in scientometric research due to their powerful data analysis and visualization capabilities, providing a comprehensive understanding of trends, networks, and patterns in the scientific literature.

VOSviewer is a well-established tool developed by the Center for Science and Technology Studies at Leiden University. It is specifically designed for constructing and visualizing bibliometric networks based on citation, cocitation, cooccurrence, and bibliographic coupling data. VOSviewer excels in generating detailed network maps that illustrate relationships between publications, authors, journals, institutions, and countries, among other entities. These maps provide a visual representation of how different research elements are interconnected, offering insights into key topics and influential studies in the field^[17–20]^.

Another critical tool in our analysis was CiteSpace, a Java-based application that combines scientometrics with data visualization techniques. CiteSpace specializes in analyzing citation networks to uncover patterns of knowledge dissemination and evolution. By applying advanced algorithms for data mining, CiteSpace helps visualize the structure and dynamics of scientific knowledge, identifying major breakthroughs, emerging research trends, and areas of intense scholarly activity. This tool is instrumental in mapping the intellectual development of specific fields and pinpointing influential studies that have shaped the direction of research^[18,21,22]^.

Additionally, we utilized Biblioshiny, an intuitive web-based application for the Bibliometrix R software. Biblioshiny is user-friendly and designed to facilitate comprehensive bibliometric analysis. It enables researchers to perform a variety of tasks, including network analysis, descriptive statistics, and visualization of bibliometric networks. This tool allowed us to conduct a detailed exploration of the dataset, uncovering trends in publication patterns, author collaborations, and thematic evolution^[23]^.

By combining these three tools, we were able to perform a multifaceted analysis of the selected literature, gaining valuable insights into the development and future directions of the research field.

## Results

### Publication trend

Trends in research within the field of IBD and emotional factors can be observed by examining the number of publications over the decades. Early research efforts were limited, with scattered publications from 1940 through the early 1960s, typically recording one or no papers annually. A noticeable shift occurred from the mid-1980s, with publication numbers slowly increasing. By 1990, annual publications reached four, and the trend continued with steady growth into the early 2000s.

**Figure 2.**
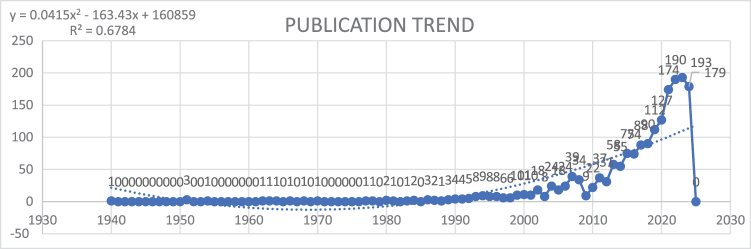
Trends in publication regarding inflammatory bowel disease and emotional factors.

The field saw a significant increase in research output starting in the 2010s, with 75 publications in 2015, growing further to 174 in 2021. This surge reflects the growing scientific interest in understanding the intersection between emotional factors and IBD. From 2020 to 2023, annual publications peaked, with 193 papers recorded in 2023. However, a minor decline was observed in 2024, with 179 publications likely reflecting publication lags typical for ongoing years (Fig. 2). This analysis highlights an expanding research focus, revealing both the historical evolution and future potential in this important interdisciplinary domain.

**Figure 3.**
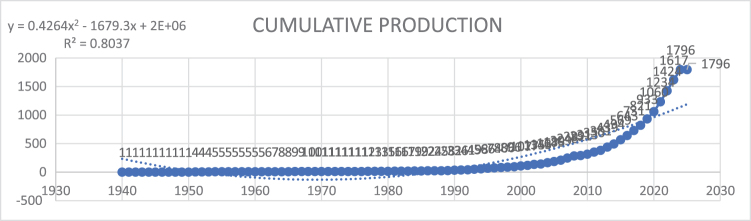
Cumulative publications regarding inflammatory bowel disease and emotional factors.

The cumulative research output in IBD and emotional factors has demonstrated a marked upward trend over the years. Early growth was slow, with cumulative publications remaining below 10 until the late 1960s. From 1970 through the mid-2000s, there was a gradual increase, reaching a cumulative total of 209 by 2006. However, the pace of research significantly accelerated from 2007 onward, driven by growing interest and advancements in the field. By 2015, the cumulative total had climbed to 569, and the rate of publication surged even further in subsequent years. Between 2016 and 2024, cumulative publications skyrocketed from 643 to 1796, reflecting the burgeoning focus on the psychological and emotional dimensions of IBD research. This substantial growth emphasizes the evolving landscape of the field and its expanding scientific impact (Fig. 3).

### Countries and institutions

**Figure 4.**
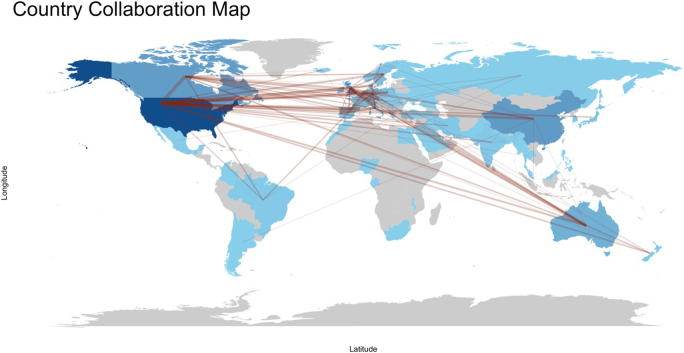
Countries collaboration in the field of inflammatory bowel disease and emotional factors.

The analysis of global contributions in the field of IBD and emotional factors identified research collaborations across 74 countries (Fig. 4). Among the top 10 contributors by publication volume, the United States led with 521 publications, demonstrating a dominant influence in the domain. England ranked second with 187 publications, closely followed by China with 186 and Canada with 185. Germany contributed 114 papers, while Australia added 109. Other prominent contributors included Italy (97), the Netherlands (64), Spain (55), and Sweden (46), highlighting significant global research engagement in this area (Table 1).

**Table 1:** Top 10 countries by number of publications

Rank	Country	No. of publications
1	United States	521
2	England	187
3	People’s Republic of China	186
4	Canada	185
5	Germany	114
6	Australia	109
7	Italy	97
8	Netherlands	64
9	Spain	55
10	Sweden	46

**Figure 5.**
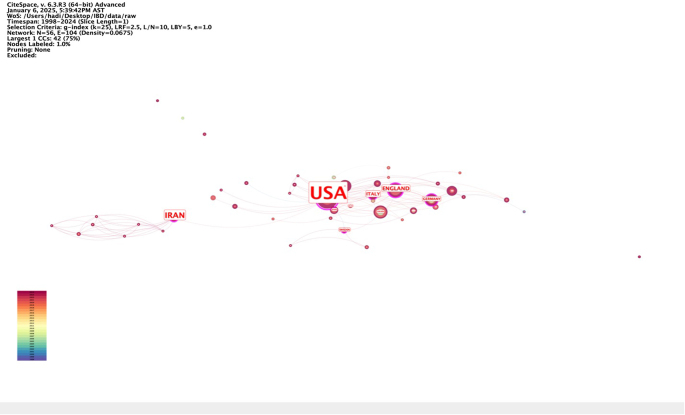
Countries with the high centrality in the field of inflammatory bowel disease and emotional factors.

**Figure 6.**
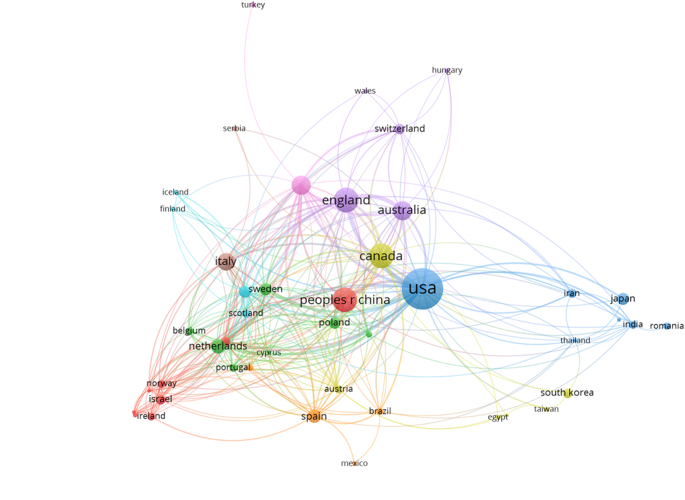
Network visualization of the countries in the field of inflammatory bowel disease and emotional factors.

Table 2 presents the top 10 countries ranked by centrality in the research network on IBD and emotional factors. The United States exhibited the highest centrality value (0.72), indicating its pivotal role in global research collaboration. Iran followed with a centrality of 0.32, while England (0.21) and Italy (0.19) also demonstrated substantial influence. Germany (0.14) and Sweden (0.11) contributed notably to network cohesion. Canada and Spain each recorded a centrality of (0.07), with Qatar (0.05) and Australia (0.02) completing the top 10. Figures 5 and 6 visualize the collaborative network among countries, emphasizing the significant roles of the United States and Iran as central nodes driving research connectivity and collaboration strength.

**Table 2:** Top 10 countries by centrality

Rank	Country	Centrality
1	United States	0.72
2	Iran	0.32
3	England	0.21
4	Italy	0.19
5	Germany	0.14
6	Sweden	0.11
7	Canada	0.07
8	Spain	0.07
9	Qatar	0.05
10	Australia	0.02

**Figure 7.**
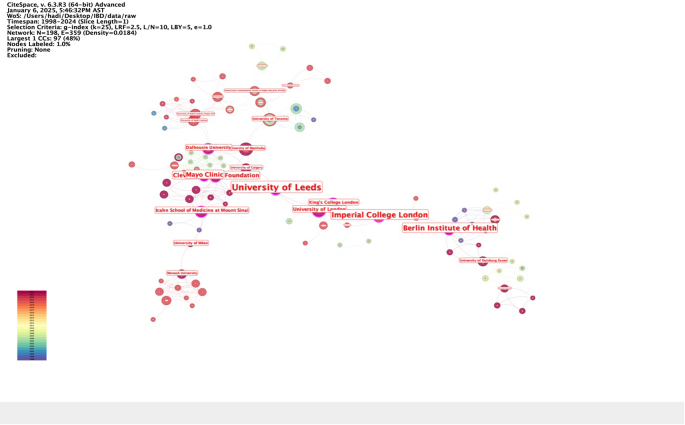
Institutions with the high centrality in the field of inflammatory bowel disease and emotional factors.

**Figure 8.**
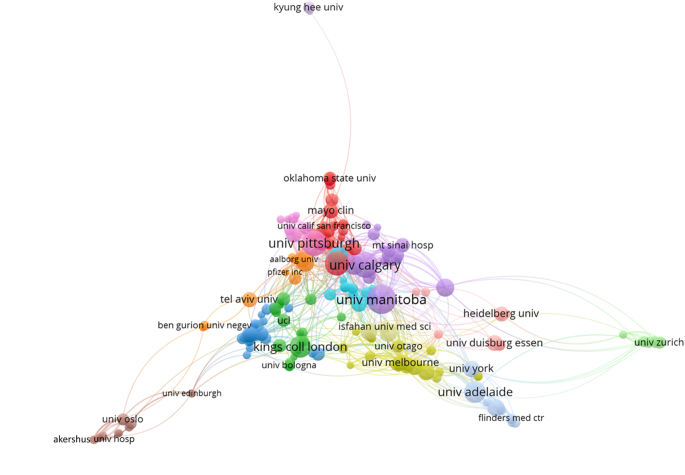
Network visualization of the institutions in the field of inflammatory bowel disease and emotional factors.

In terms of institutional contributions to research on IBD and emotional factors, the University of Manitoba led with 67 publications. The University of Calgary followed with 51, while the University of Pittsburgh contributed 49 papers. The University of North Carolina and the University of Toronto produced 45 and 37 publications, respectively. The University of Adelaide and King’s College London each accounted for 36 publications. Other notable institutions included Dalhousie University^[24]^, the Icahn School of Medicine at Mount Sinai^[25]^, and McMaster University^[26]^ (Table 3 and Fig. 7). Figure 8 illustrates the collaboration strength among these institutions, highlighting their interconnected roles in advancing the field.

**Table 3:** Top 10 institutions by number of publications

Rank	Institutions	No. of publications
1	University of Manitoba	67
2	University of Calgary	51
3	University of Pittsburgh	49
4	University of North Carolina	45
5	University of Toronto	37
6	University of Adelaide	36
7	King’s College London	36
8	Dalhousie University	29
9	Icahn School of Medicine at Mount Sinai	26
10	McMaster University	25

### Journals and cocited journals

**Figure 9.**
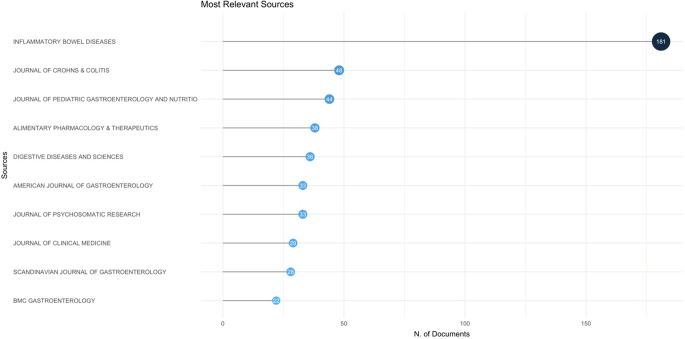
Top 10 journals in the field of inflammatory bowel disease and emotional factors.

The bibliometric analysis revealed a total of 560 journals focused on IBD, with *Journal of Crohn’s & Colitis* being the leading publication, contributing 48 articles. *Journal of Pediatric Gastroenterology and Nutrition* followed closely with 44 publications, while *Alimentary Pharmacology & Therapeutics* had 38, and *Digestive Diseases and Sciences* accounted for 36. Other notable journals included *American Journal of Gastroenterology* (33 articles), *Journal of Psychosomatic Research* (33 articles), *Journal of Clinical Medicine* (29 articles), *Scandinavian Journal of Gastroenterology* (28 articles), and *BMC Gastroenterology* (22 articles). *World Journal of Gastroenterology* published 22 articles, while *Clinical Gastroenterology and Hepatology* and *Gut* both had 21. Figure 9 illustrates the top 10 journals in the field of IBDs, highlighting their contributions to this research area.

**Figure 10.**
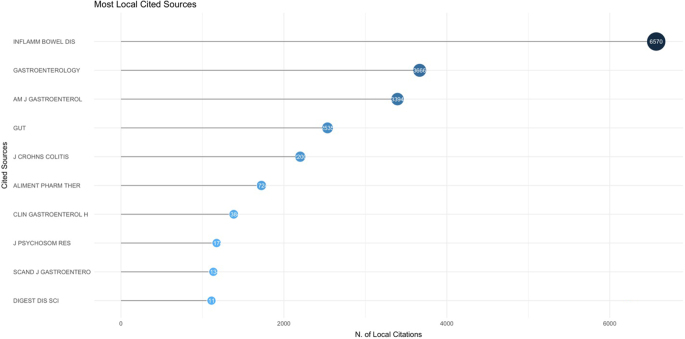
Top 10 cocited journals in the field of inflammatory bowel disease and emotional factors.

The cocitation analysis of journals in the field of IBD and emotional factors revealed that *Inflammatory Bowel Diseases* had the highest number of citations, with 6570 citations. It was followed by *Gastroenterology* with 3666 citations and *American Journal of Gastroenterology* with 3394 citations. *Gut* received 2535 citations, while *Journal of Crohn’s and Colitis* garnered 2200 citations. Additional prominent journals included *Alimentary Pharmacology & Therapeutics* with 1724 citations and *Clinical Gastroenterology and Hepatology* with 1384 citations. Figure 10 illustrates the top 10 cocited journals in the field of IBD and emotional factors.

**Figure 11.**
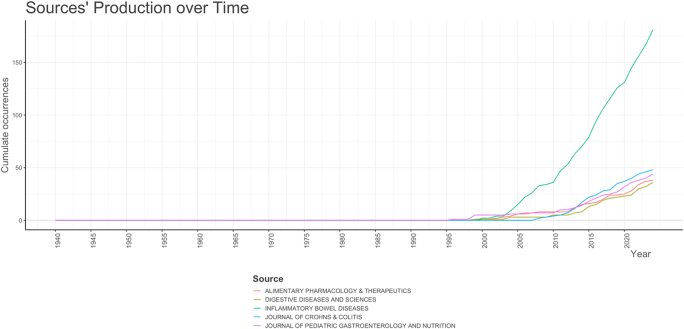
Journals’ productions over time in the field of inflammatory bowel disease and emotional factors.

The analysis of publication trends over time in the field of IBD and emotional factors, depicted in Figure 11, reveals significant shifts in research output across various journals. The cumulative number of publications remained low until around 2000, with few contributions from key journals such as *Journal of Crohn’s and Colitis* and *Journal of Pediatric Gastroenterology and Nutrition*. From 2000 onward, there was a steady increase in publications, with journals like *Alimentary Pharmacology & Therapeutics* and *Digestive Diseases and Sciences* beginning to contribute more significantly. The most notable surge occurred after 2015, particularly in journals such as *Inflammatory Bowel Diseases*, which saw a dramatic rise in publications. This growth was most pronounced after 2020, with an accelerating trend reflecting growing interest and advancements in the study of emotional factors in IBD. The overall trend points to a marked increase in research output over the past two decades, with an especially rapid growth in recent years, highlighting the expanding recognition of the role emotional factors play in IBD.

### Authors and cocited authors

The analysis of contributions in the domain of IBD and emotional factors identified several influential authors who have shaped the research landscape. Charles N. Bernstein emerged as the most prolific author, contributing the highest number of publications at 55. Following him, Lesley A. Graff produced 39 publications, while Antonina Mikocka-Walus ranked third with 32 publications. Laurie Keefer and Michael D. Kappelman were also notable contributors, with 30 and 26 publications, respectively (Table 4).

**Table 4:** Top 10 authors by publications, citations, and cocitations

Number	Author with a high number of publications	No. of publications	Author with a high number of citations	No. of citations	The most cocited authors	No. of cocitations
1	Bernstein, Charles N.	55	Bernstein, Charles N.	3223	Mikocka-Walus, A	525
2	Graff, Lesley A.	39	Walker, John R.	2276	Bernstein, Cn	495
3	Mikocka-Walus, Antonina	32	Graff, Lesley A.	2255	Graff, La	456
4	Keefer, Laurie	30	Ananthakrishnan, Ashwin N.	1941	Drossman, Da	412
5	Kappelman, Michael D.	26	Mikocka-Walus, Antonina	1522	Ananthakrishnan, An	373
6	Andrews, Jane M.	25	Ford, Alexander C.	1499	Gracie, Dj	371
7	Ford, Alexander C.	25	Miller, Norine	1468	Levenstein, S	299
8	Long, Millie D.	25	Keefer, Laurie	1158	Knowles, Sr	287
9	Marrie, Ruth Ann	23	Clara, Ian	1041	Szigethy, E	282
10	Szigethy, Eva	23	Rawsthorne, Patricia	1008	Zigmond, As	262

In terms of research impact, Charles N. Bernstein again led with the highest number of citations, totaling 3223. John R. Walker followed with 2276 citations, and Lesley A. Graff made a significant impact with 2255 citations. Ashwin N. Ananthakrishnan and Antonina Mikocka-Walus completed the top five, with 1941 and 1522 citations, respectively (Table 4).

The cocitation analysis, which reveals influential collaborations and shared references, identified Mikocka-Walus, A., as the most cocited author with 525 citations. Charles N. Bernstein closely followed with 495 cocitations, and Lesley A. Graff ranked third with 456. David A. Drossman and Ashwin N. Ananthakrishnan also featured prominently in the cocitation network, with 412 and 373 cocitations, respectively (Table 4).

### Top cited papers

Table 5 presents the top 10 most cited papers focusing on the interplay between IBD and emotional factors, demonstrating their substantial influence on this research area. The most cited paper, titled “Epidemiology and risk factors for IBD,” published in 2015, has accumulated 1153 citations, reflecting its foundational impact on understanding the condition’s prevalence and contributing factors. The second most cited work, “Development of the perceived stress questionnaire: a new tool for psychosomatic research,” published in 1993, has garnered 667 citations, showcasing its significance in psychosomatic research and stress assessment.

**Table 5:** Top 10 cited papers in the field of inflammatory bowel disease and emotional factors

Number	Title of the most cited paper	Published year	No. of citations
1	Epidemiology and risk factors for IBD^[27]^	2015	1153
2	Development of the perceived stress questionnaire: a new tool for psychosomatic research^[26]^	1993	667
3	Brain-gut interactions in inflammatory bowel disease^[25]^	2013	447
4	Psychological stress in IBD: new insights into pathogenic and therapeutic implications^[28]^	2005	430
5	Quality of life in inflammatory bowel disease in remission: the impact of IBS-like symptoms and associated psychological factors^[29]^	2002	409
6	Psychological stress and corticotropin-releasing hormone increase intestinal permeability in humans by a mast cell-dependent mechanism^[24]^	2014	407
7	Impact of depressive mood on relapse in patients with inflammatory bowel disease: a prospective 18-month follow-up study^[30]^	2004	400
8	Controversies revisited: a systematic review of the comorbidity of depression and anxiety with inflammatory bowel diseases^[31]^	2016	386
9	Depression and anxiety in patients with inflammatory bowel disease: a systematic review^[32]^	2016	363
10	Stress and exacerbation in ulcerative colitis: a prospective study of patients enrolled in remission^[33]^	2000	331

In third place, “Brain-gut interactions in inflammatory bowel disease,” published in 2013, has received 447 citations, highlighting its role in elucidating the complex neurogastroenterological connections in IBD. Fourth on the list, the 2005 study “Psychological stress in IBD: new insights into pathogenic and therapeutic implications” amassed 430 citations, emphasizing the critical role of psychological stress in disease progression and management strategies.

The fifth-ranked paper, “Quality of life in inflammatory bowel disease in remission: the impact of IBS-like symptoms and associated psychological factors,” published in 2002, has been cited 409 times, focusing on the persistent quality-of-life challenges faced by patients. “Psychological stress and corticotropin-releasing hormone increase intestinal permeability in humans by a mast cell-dependent mechanism,” from 2014, ranks sixth with 407 citations, shedding light on stress-induced physiological responses in IBD.

The seventh most cited paper, “Impact of depressive mood on relapse in patients with inflammatory bowel disease: a prospective 18-month follow-up study,” published in 2004, has 400 citations, emphasizing the influence of mood disorders on disease relapse. At eighth place, “Controversies revisited: a systematic review of the comorbidity of depression and anxiety with inflammatory bowel diseases,” published in 2016, has been cited 386 times, reflecting ongoing debates and findings on the mental health burden in IBD patients.

**Figure 12.**
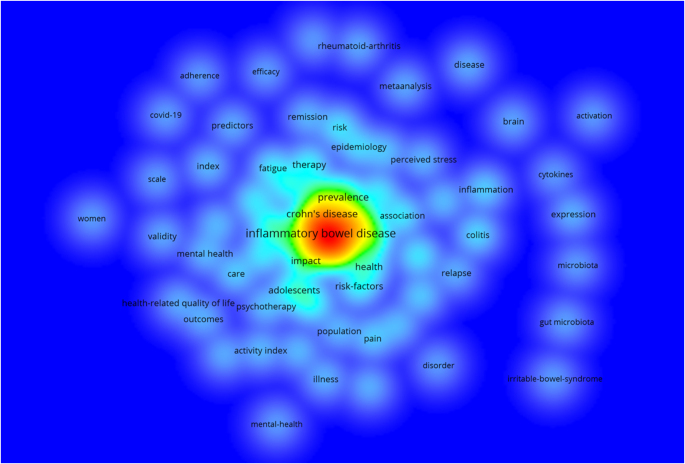
Density visualization map of the keywords.

“Depression and anxiety in patients with inflammatory bowel disease: a systematic review,” also from 2016, ranks ninth with 363 citations, underlining the prevalence of mental health challenges in this population. Finally, the tenth most cited study, “Stress and exacerbation in ulcerative colitis: a prospective study of patients enrolled in remission,” published in 2000, has accrued 331 citations, illustrating the link between stress and symptom flare-ups. Figure 12 provides a visualization of citation bursts, showing key trends and emerging focus areas in the research landscape since 2015.

### Keyword trends, hotspots, cluster analysis

**Figure 13.**
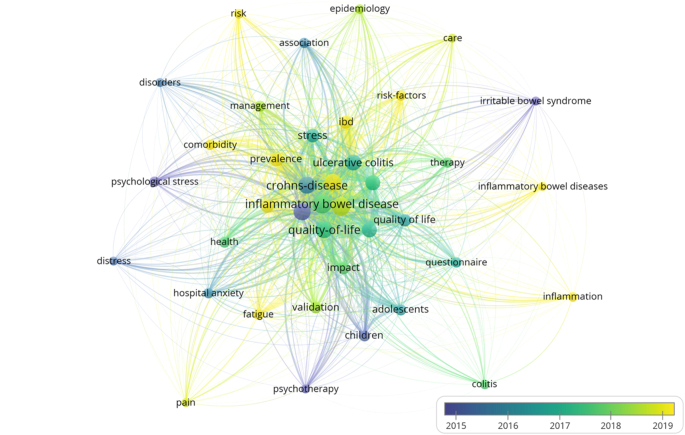
Overlay visualization keywords.

The analysis of keywords revealed several frequently recurring terms in research related to IBD and emotional factors. The 10 most prominent keywords were “inflammatory bowel disease” (*n* = 743), “depression” (*n* = 683), “crohn’s disease” (*n* = 558), “anxiety” (*n* = 543), “quality of life” (*n* = 533), “ulcerative colitis” (*n* = 515), “ulcerative colitis” (variant) (*n* = 387), “inflammatory-bowel-disease” (variant) (*n* = 370), “crohn’s disease” (variant) (*n* = 348), and “stress” (*n* = 268) (Fig. 13).

## Discussion

The bibliometric analysis conducted in this study provides a comprehensive overview of the evolution of research on IBD and its intersection with emotional factors. Our findings suggest a substantial growth in research output over the past two decades, with significant increases observed in the 2010s. This surge can likely be attributed to growing awareness of the psychosomatic aspects of chronic diseases and increasing recognition of the impact of emotional health on disease progression and management.

In terms of global contributions, countries like the United States, the United Kingdom, and China dominate the research landscape. The United States consistently leads the publication volume totally, and Canadian institutions such as the University of Manitoba and the University of Calgary are emerging as key players. This may be due to robust funding for medical research and a long-standing emphasis on patient-centered care, particularly in gastrointestinal disorders. It is noteworthy that while Western countries remain at the forefront, emerging research hubs in countries like Iran and India are becoming increasingly visible, possibly due to enhanced research infrastructure and international collaborations.

The journal landscape reveals that leading journals such as *Journal of Crohn’s & Colitis* and *Gastroenterology* are central to the dissemination of research on IBD. These journals not only provide a platform for clinical studies but are also critical in advancing interdisciplinary research that links IBD to psychosocial factors. This highlights the increasing importance of integrated approaches to disease management, including addressing the emotional and psychological needs of patients. Furthermore, the cocitation analysis indicates that journals such as *Inflammatory Bowel Diseases* and *Digestive Diseases and Sciences* serve as critical nodes in research networks, influencing the direction of the field.

Several key authors, such as Charles N. Bernstein, have made substantial contributions to the research on the relationship between emotional well-being and IBD. Their work has shaped current clinical guidelines that recognize the importance of managing mental health alongside the physical symptoms of IBD. Bernstein’s research on the psychological impacts of IBD, including depression and anxiety, has been fundamental in bridging the gap between gastrointestinal care and mental health support^[25]^.

Additionally, there is a need for more studies on interventions that target both IBD and emotional health simultaneously. While individual therapies for IBD or psychological disorders are well-established, there is less focus on integrated models of care^[34]^. Mindfulness-based interventions have shown promising results for patients with IBD. Multiple studies have demonstrated significant improvements in psychological well-being, including reduced anxiety and depression, and enhanced quality of life^[35–38]^.

Moreover, emotional factors such as anxiety and depression have been shown to worsen disease outcomes, increase disease activity, and contribute to lower quality of life in IBD patients^[39,40]^. Studies like those by K. Skonieczna-Żydecka *et al*^[41]^ highlight the intricate communication within the gut–brain axis (GBA) and its profound impact on gastrointestinal and mental health. The study emphasizes the role of gut microbiota in shaping brain regions associated with emotions, cognition, and physical activity, paving the way for innovative diagnostic tools and microbiota-based therapies. This underscores the importance of integrating microbiome-focused approaches in managing GBA-related disorders to improve therapeutic outcomes.

## Limitations

This study has several limitations that should be acknowledged. First, the analysis relied exclusively on data from Web of Science, which may have excluded relevant publications indexed in other databases, potentially limiting the scope of the results. Additionally, the inclusion of only English-language articles introduces a language bias, potentially overlooking valuable research in other languages. Bibliometric indicators such as citation counts may be influenced by factors unrelated to research quality, including journal reputation and geographical citation patterns. Furthermore, while the keyword strategy was comprehensive, studies using alternative terminology or related topics may have been inadvertently excluded. The interdisciplinary nature of the topic introduces challenges in standardizing bibliometric parameters, and the analysis does not delve into the specific content or quality of the studies reviewed. Lastly, as a static snapshot, the findings cannot fully capture the dynamic and ongoing nature of research in this area. Despite these limitations, the study provides valuable insights into the research landscape, offering a foundation for future investigations.

## Conclusion

This bibliometric analysis highlights the growing recognition of the intricate relationship between emotional factors and IBD, reflecting a rapidly expanding body of research over recent decades. From the historical underrepresentation of psychological considerations in IBD management to the current surge in publications, it is evident that emotional and psychological factors are now central to understanding and addressing the challenges faced by individuals living with IBD.

The analysis revealed significant contributions from leading countries, institutions, authors, and journals, underscoring a strong global commitment to advancing this interdisciplinary field. While the United States emerged as the dominant leader in research output and collaboration, other countries like England, Canada, and China demonstrated growing influence and engagement. Similarly, institutions such as the University of Manitoba and the University of Calgary have established themselves as pivotal hubs of research in this domain.

Key trends in publication outputs and the increasing prominence of collaborative networks point to the evolving nature of this research field, driven by the need to integrate gastroenterological and psychological care. Influential studies, high-impact journals, and prominent authors have paved the way for a deeper understanding of the bidirectional relationship between psychological distress and IBD progression. However, this analysis also revealed critical research gaps, such as the need for longitudinal studies, a focus on diverse patient populations, and investigations into culturally specific psychological interventions.

Future research should aim to explore emerging areas such as personalized interventions addressing emotional and psychological factors, the role of digital health tools, and the long-term impact of integrated care approaches. Enhanced collaboration between gastroenterologists, psychologists, and researchers from allied disciplines will be essential to address the complex interplay of emotional and physical health in IBD.

By identifying historical trends, current advances, and future directions, this study provides a roadmap for researchers, clinicians, and policymakers seeking to improve the quality of life and clinical outcomes for patients with IBD through a holistic and multidisciplinary approach.

## Data Availability

The datasets used and/or analyzed during the current study are accessible from the corresponding author on reasonable request.

## Research registration unique identifying number (UIN)

The study is a bibliometric study and UIN is not applicable.
